# Proactive Prediction of Alarms in Hemodialysis Machines: A Comparative Evaluation of AI Models for Clinical Prognostic Support

**DOI:** 10.3390/diagnostics16193095

**Published:** 2026-09-24

**Authors:** Alessia Nicosia, Nunzio Cancilla, Marco Siino, Michele Passerini, Francesca Sau, Ilenia Tinnirello, Andrea Cipollina

**Affiliations:** 1 Dipartimento di Ingegneria, Università degli Studi di Palermo, Viale delle Scienze Ed. 6, 90128 Palermo, PA, Italy; alessia.nicosia@unipa.it (A.N.); ilenia.tinnirello@unipa.it (I.T.); andrea.cipollina@unipa.it (A.C.); 2 Dipartimento di Ingegneria Elettrica, Elettronica e Informatica, Università di Catania, Via Santa Sofia 64, 95123 Catania, CA, Italy; marco.siino@unict.it; 3 Mozarc Medical^®^, Via Camurana 1, 41037 Mirandola, MO, Italy

**Keywords:** artificial intelligence, alarm prediction, dialysis systems, patient safety, clinical support, kidney disease

## Abstract

**Background/Objectives**: Hemodialysis is a treatment for patients with kidney disease, yet it remains risk-prone due to potential technical or physiological instabilities. Dialysis machines rely on reactive safety mechanisms that trigger alarms only when predefined thresholds are reached. This often alerts clinical staff only after a complication has already occurred, contributing to potential delays in interventions. This study aims to develop a proactive prognostic framework capable of anticipating system instabilities before they transform into clinical adverse events. **Methods**: This work proposes a predictive approach for the proactive detection of patient- or process-related alarm conditions using ten machine learning models and one deep learning architecture. A dataset consisting of over two million records sampled at 0.5 s intervals from 95 dialysis sessions was assembled by merging machine logs of sensor readings and the binary machine status (alarm/normal). **Results**: Among the models tested, Random Forest emerged as the most robust predictive tool, achieving an F_1_ score higher than 0.85 for predicting alarms up to one minute in advance, with precision (~0.83), recall (~0.87), specificity (~0.99), AUROC (~0.99), and AUPRC (~0.94) values indicating strong performance, and stable performance across validation and test sets. A progressive decrease in performance was observed for longer prediction time windows. **Conclusions**: These findings demonstrate the feasibility of predicting dialysis-machine-generated alarms in advance using machine learning and deep learning models. Providing advance warning could give clinicians the opportunity to anticipate impending alarm conditions and, where appropriate, implement timely corrective actions, potentially improving treatment management and the operational reliability of renal replacement therapies.

## 1. Introduction

One of the major health problems in modern society is renal insufficiency, a condition in which blood flow to the kidneys is reduced due to renal artery disease. This impairment in kidney function affects more than 7 million people worldwide [1], for whom the only life-saving treatments available are renal replacement therapies or kidney transplantation.

In this context, the present section provides the background and motivation for the study presented in this manuscript. Specifically, it first presents an overview of renal replacement therapies and their operating principles, then reviews recent research on the application of Artificial Intelligence (AI) in dialysis, and finally introduces the aims and scope of the work, emphasizing its novelty and expected impact within the broader context of predictive healthcare.

### 1.1. Renal Replacement Therapies: A General Overview

Renal replacement therapies represent the standard treatment modalities for patients with kidney failure and primarily include hemodialysis (HD), hemofiltration (HF), and hemodiafiltration (HDF).

HD is a therapy that uses a semipermeable membrane to separate two fluids: the patient’s blood, which contains toxins that the kidneys can no longer effectively remove, and a solution rich in electrolytes and glucose called dialysate. Through diffusion across the membrane, low-molecular-weight solutes (up to ~500 Da) such as urea and creatinine are eliminated, while essential components, including proteins and blood cells, are retained [2].

There are variants of hemodialysis, including HF and HDF, which differ in the mechanisms they use to remove waste products and excess fluids from the blood. HF relies solely on the convective removal of uremic toxins. Unlike standard hemodialysis, HF does not use dialysate; instead, it employs a pressure gradient to filter out excess fluids and solutes with medium to high molecular weights (in the range of 500 to 15,000 Da). To maintain stable blood volume and electrolyte balance, replacement fluids are introduced either before (pre-dilution) or after (post-dilution) the blood passes through the filtration unit. HDF combines the mechanisms of both hemodialysis and hemofiltration, allowing for the efficient removal of fluids and solutes across a broad range of molecular weights (larger than 15,000 Da). This dual approach more closely replicates the kidneys’ natural filtration function, making HDF the most widely used technique in renal care [3].

Despite the differences among the three above-mentioned membrane-based blood purification techniques, the term “hemodialysis” will be used throughout the paper to refer to all three treatments interchangeably.

The semipermeable membrane is housed inside hemodialyzers, cylindrical modules typically a few centimeters in diameter and about 20–30 cm in length [4]. The hemodialyzers work within dialysis machines, which are sophisticated systems managed by trained medical staff in a hospital setting. All dialysis machines share some essential features, including a wide range of sensors and pumps that precisely control and monitor the treatment process. Among the monitored variables, particular attention is given to key parameters such as blood pressure, protein retention, and urea clearance efficiency, the latter being a crucial indicator of dialysis performance [5].

Figure 1 reports a schematic of the dialysis circuit along with indications of the main sensors in the machine.

Nevertheless, dialysis treatment carries several risks, resulting from technical issues or sudden changes in the patient’s condition. To reduce these risks, modern dialysis systems are complex cyber-physical systems equipped with an array of sensors monitoring pressure, flow, and temperature to ensure the safety and efficacy of the treatment. Advanced control systems and alarm mechanisms notify medical personnel and can automatically interrupt the procedure if necessary [6,7]. Currently, safety in dialysis is managed through a reactive paradigm. Alarms are triggered by anomalies such as abnormal blood pressure, temperature fluctuations, irregular heart rate, blood leakage, or malfunctions in the machine’s ultrafiltration, degassing, or fluid delivery systems. However, one limitation of these safety systems is that, although they are designed with intervention margins to protect the patient, they are still triggered only once predefined physiological or systemic thresholds are reached. As a result, alarms often alert medical staff only after a complication has already begun to manifest. Furthermore, the high frequency of these threshold-based alerts contributes to “alarm fatigue” among clinical staff, potentially leading to delayed responses to critical events. These limitations highlight a significant gap in current medical diagnostics: the lack of proactive tools with prognostic capabilities for anticipating instabilities before they reach a critical threshold. The need for more advanced monitoring and predictive strategies capable of anticipating potential issues before they occur is a goal that recent developments in AI have increasingly aimed to address.

### 1.2. Artificial Intelligence in Hemodialysis

Since the last century, the use of AI techniques and its subfields, Machine Learning (ML) and Deep Learning (DL), has sparked a paradigm shift in biomedical engineering [8], by enabling the analysis of complex, non-linear datasets that characterize modern healthcare.

Within the broader field of renal medicine, AI-based approaches have been increasingly explored for different diagnostic and predictive applications. In this regard, Neha and Bansal [9] have recently proposed a radiology–radiomics fusion-based deep learning framework for the non-invasive classification of malignant renal cell carcinoma subtypes, combining radiomic and deep imaging features to support automated diagnostic discrimination. This study provided a representative example of the growing use of AI-based methods in kidney-related applications, achieving a validation accuracy of 96.70% and a test accuracy of 97.3% for the classification of three major renal cell carcinoma subtypes.

Focusing specifically on hemodialysis, recent research has predominantly focused on patient-centered prognostic models designed to predict acute intradialytic complications, with the aim of optimizing clinical outcomes, particularly in patients whose health is severely compromised. Among the most frequent challenges during dialysis sessions are hemodynamic instability, such as fluctuations in blood pressure and fluid volume, as well as adverse effects including muscle cramps and nausea. These complications can significantly impact both treatment efficacy and the patient’s overall health status, highlighting the need for proactive, data-driven strategies. In this context, Ren et al. [10] analyzed over 67,000 dialysis sessions using ten different ML algorithms for predicting intradialytic hypotension and intradialytic hypertension, achieving high predictive performance (Area Under the Curve, AUC, up to 0.89) for both conditions. With a more sophisticated approach, Li et al. [11] predicted the same parameters employing an explainable model, which is useful for interpreting the influence of clinical variables. Specifically, using 46,000 dialysis sessions of 193 patients, the authors achieved an overall accuracy exceeding 85% and determined that blood pressure before dialysis, age, and difference between the sodium levels in the blood and in the dialysis fluid were the most important risk factors. While these models achieve high predictive accuracy, they often rely on static clinical variables or low-frequency physiological data, which may fail to capture the rapid, transient fluctuations within the extracorporeal circuit that precede clinical instability.

Other applications have addressed specific dialysis-related diagnostic challenges, such as assessing vascular health [12] or fluid status [13].

For example, Zhang et al. [12] developed sophisticated deep neural networks to analyze the sounds produced by Arteriovenous Fistulas (AVFs) in hemodialysis patients with the goal of detecting stenosis, a narrowing of the blood vessel that can lead to serious complications. The model was trained on sound recordings from over 800 patients and used visual representations of these sounds to identify patterns linked to healthy or narrowed fistulas. Thanks to a combination of advanced techniques, the system identified cases of stenosis with 99% accuracy, outperforming previous similar approaches [14,15] and improving vascular access management.

Niel et al. [13] developed a multi-layer neural network to support dry weight assessment, a process that traditionally relies on clinical judgment and can be highly variable. Their model integrated multiple clinical indicators and outperformed experienced nephrologists in most cases.

Another use of AI algorithms in the dialysis field regards the prediction of the outcome of the therapy, days, months, or years later, with the aim of analyzing patients’ clinical conditions and anticipating possible future complications, up to death. For example, Motofelea et al. [16] conducted a systematic review comparing various ML models for predicting mortality in hemodialysis patients. The study evaluated models such as Logistic Regression (LR), Extreme Gradient Boosting (XGBoost), and Random Forest (RF), showing that AI-based approaches can closely approximate real-world mortality rates. Among these, the XGBoost model estimated a mortality rate of 9.93%, compared to an observed rate of 13.73%, highlighting the potential of AI models to support early identification of high-risk patients.

Furthermore, the prediction of distressing symptoms, such as emesis during hemodialysis, a complication that can lead to early termination of the session and reduced treatment efficacy, has been explored by Chen et al. [17]. Using data from 281 patients and applying a regression model, parameters such as sex, height, heart rate, lymphocyte count, and lactate dehydrogenase levels were identified as the most relevant predictors of this condition, potentially supporting the implementation of timely preventive strategies.

However, a common limitation in these studies is their focus on the biological outcome rather than the dialysis machine that mediates the treatment. By the time a patient-centered symptom (like vomiting or hypotension) is predicted or detected, the window for simple preventive adjustment may already be closing. A distinct branch of research focuses on system-oriented monitoring. In a recent paper, Xie et al. [18] proposed a neural network model for the visual recognition of alarm lights on dialysis machines. It offered a non-invasive method for real-time monitoring of machine status, indirectly contributing to the prevention of vascular complications by enabling early detection of operational anomalies. However, this study focuses on real-time alarm recognition, which differs from the predictive maintenance perspective explored, for example, by Rahman et al. [19]. After comparing several ML algorithms with a DL model, the ensemble classifier achieved the best performance in optimizing the utilization of critical medical devices and minimizing their downtime. The model analyzed data, collected by fifteen hospitals in Malaysia, from 8,294 medical devices including multiparametric monitors, ventilators, diagnostic imaging equipment, life support systems, and infusion pumps. The study evaluated mechanical, electronic, calibration, system, and power failures, predicting the probability of device failure within three years with an accuracy of up to 79.50%, a significant achievement considering the diversity and complexity of the devices and potential failure modes.

While innovative, these approaches remain reactive, as they merely automate the recognition of an alarm that has already been triggered. This highlights a critical gap in the literature: the lack of systems capable of leveraging high-frequency machine telemetry to anticipate these alarms before they reach a critical threshold.

### 1.3. Aim of the Work

Current dialysis safety mechanisms are hindered by threshold-based logic, which contributes to “alarm fatigue” and delayed intervention. The present study addresses this scientific gap in clinical alarm prediction in dialysis, by treating alarms as early indicators of patient- or process-related complications during treatment. By utilizing a dataset of over two million records sampled at a high resolution of 0.5 s, the proposed framework is conceived as a prognostic-support tool. It was designed to anticipate the possible onset of such events by exploring different model families and prediction time spans, with the aim of providing operators with earlier information that could support timely intervention and the safe continuation of therapy.

The proactive prediction of alarms remains underexplored [20]. This approach distinguishes itself from the existing literature by treating machine-generated alarms not as static events to be filtered or recognized, but as predictable states that can potentially be anticipated with a clinically meaningful lead time. This methodological approach may also be applicable to a wide range of clinical and industrial monitoring scenarios, such as intensive care monitoring, mechanical ventilation, cardiac arrhythmia detection, or extracorporeal support systems [21,22]. These perspectives highlight the potential broader relevance and versatility of the proposed framework beyond the specific application investigated, with the need for further prospective validation to demonstrate its actual clinical benefit.

## 2. Methods

This section describes the methodological framework adopted in this study. Specifically, it is structured into three main parts, including the description of the dataset, the pre-processing and feature selection procedures, and the implementation and evaluation of the AI models developed for the prediction task, from which the best-performing model was selected for further analysis.

### 2.1. Description of the Dataset

Data from chronic dialysis therapies performed with hemodialysis equipment were used. All treatments were delivered using five dialysis machines of the same model, manufactured by Mozarc Medical^®^ (Modena, Italy) and used for extracorporeal renal replacement therapy in adult patients under routine clinical practice. Specifically, this equipment includes pressure transducers monitoring arterial, venous, pre-filter, and transmembrane pressures; flow and pump encoders that measure dialysate delivery, ultrafiltration, and blood-pump activity; conductivity and temperature sensors assessing dialysate composition and thermal balance; as well as safety sensors such as air and foam detectors and venous-clamp status indicators.

In a regular working day, considering the time between 8 a.m. and 8 p.m., each machine performs a maximum of two treatments per day generating for each session a set of ten files identified by a unique ID code. These files provide detailed information on the treatment parameters selected for the patient, the machine’s operating phases during the dialysis session, and the quantities measured by the sensors monitoring the process. They do not contain any patient-specific information, such as gender, age, medical history, or identifying details which, even if available, would not have been relevant for the aim of our study, which focuses on detecting deviations in machine-monitored parameters rather than optimizing individual treatments. In addition, as the files reflect only the chronological order of treatments performed, without distinguishing whether multiple sessions belong to the same patient, in this study data were grouped by the date of the treatment rather than by individual patient.

For the implementation and evaluation phase, four months of therapies were considered, all performed using the same dialysis machine model to ensure a consistent acquisition protocol across the treatments.

Python^®^ 3.11 and its scientific libraries were used for data processing and model development. Namely, “NumPy” (version 1.26.4) and “pandas” (version 2.2.3) for representing the input data in vectorial form, “scikit-learn” (version 1.5.2) for the training phase, and “Matplotlib” (version 3.9.2) and “seaborn” (version 0.13.2) for analyzing the results. The models were implemented on a workstation equipped with an Intel^®^ Core^TM^ i7-6700 CPU and 32 GB of DDR4 RAM (Intel Corporation, Santa Clara, CA, USA).

Training was performed on data recorded during each treatment by the same type of dialysis machine. The data were assembled into a file comprising records of 95 hemodialysis treatments which, although limited in number, cover a substantial temporal span and provide a detailed and representative description of the selected machine’s operating behavior.

In this unified dataset, each row contains measurements of the patient’s vital parameters, regular sampling of the machine’s internal parameters, records of the operator’s manual actions, and the binary machine status (alarm/normal) as recorded by the machine monitoring system. This study relies on an extended version of the dataset used in our earlier work [23], here expanded to 93 variables and 2,586,776 rows acquired every 0.5 s of treatment. Among these, the “type” and “code” columns are particularly important, as they reflect the progression of the therapy and indicate the presence or absence (“type”, binary variable) and the specific category (“code”) of any patient- or process-related issues, such as abnormal flow rates, deviations in body temperature or heart rate, blood loss, and other physiological or systemic abnormalities. Although our predictive model focuses exclusively on the prediction of the binary information provided by the “type” column, Table 1 reports the main alarm monitoring systems and the corresponding conditions that trigger an alarm.

As a complementary and more detailed characterization of the binary alarm endpoint, Table S1 in Supplementary Materials reports the distribution and functional classification of all individual machine-generated alarm codes included in the dataset, providing context on the types of events considered.

In order to formalize the predictive problem, a discrete time variable *t* was introduced, defined as *t* = *n* × 0.5 s (with *n* ∈ N, where N denotes the set of natural numbers). In other words, each row in the dataset corresponds to a measurement taken every 0.5 s, so that *n* rows represent a time span of *n*/2 s. The dataset is therefore represented as a time series of 93 parameters sampled at these discrete intervals, with the last column *y*(*t*) denoting the machine’s operational status at time *t*. Consequently, each row is regarded as a complete observation of the system and is treated as a time sample of the machine parameters in the rest of the paper.

Figure 2 shows a simplified representation of a small portion of the dataset, highlighting in red the last column “type” with the binary output *y*(*t*).

### 2.2. Dataset Pre-Processing and Model Implementation

The dataset was filtered to include only the dialysis treatment phase (*dmstate* = 4), as the study focused specifically on the patient treatment process. Other operational stages of the machine, such as preparation, washing, and post-treatment cleaning, were excluded to ensure consistency and relevance for model development.

The *p*-value was calculated to assess the statistical significance of the relationships between the variables studied, whether they can be attributed to random variation [24]. The general formula that describes the *p*-value is defined as:

(1) $$p\text{-}\mathrm{value}=P(observation|H_{0}true)$$

where $H_{0}$ is the null hypothesis, observation is the actual value from the data collected in the study to compare with the null hypothesis, and p-value is the probability of obtaining the observation assuming that the null hypothesis is true. If the *p*-value is low, it means that the observation is highly unlikely under the null hypothesis, which suggests that the null hypothesis can be rejected.

Therefore, low *p*-values (usually less than 0.05) indicate that the observed result is unlikely if the null hypothesis is true. This leads to the rejection of the null hypothesis. On the contrary, high *p*-values indicate that the observed data is compatible with the null hypothesis, so there is insufficient evidence to reject it. In this work, a *p*-value < 0.05 was found for all the features, indicating statistically significant associations between the variables under investigation.

After this preliminary analysis, ten machine learning models were tested and evaluated for predicting the selected output type. To optimize training and enhance performance, hyperparameter tuning was performed for each model using “RandomizedSearchCV” [25], a method provided by “scikit-learn” in Python^®^ that evaluates multiple hyperparameter configurations by randomly sampling combinations from a predefined search space.

Each machine learning model underwent ten randomized hyperparameter trials on a development set comprising 70% of the treatment sessions, with each configuration evaluated using 5-fold cross-validation. Both the initial dataset partitioning and the cross-validation folds were defined at the treatment-session level, ensuring that all samples belonging to the same treatment were assigned exclusively to the same subset or fold. This strategy prevented information leakage between training, validation, and test data arising from the strong temporal correlation among consecutive measurements within the same treatment session [26]. In addition to these conventional models, a deep learning model was also tested using the same 5-fold cross-validation approach, adopting a setup commonly used in the literature [27] as a benchmark for comparison.

From the residual 30% of the dataset, 15% was held out as a validation set, used to independently evaluate the best configuration identified for each machine learning model and the selected deep learning model, and to assess their ability to generalize to unseen data. Finally, the remaining 15% was reserved as a final testing set and used exclusively to evaluate the selected best-performing model after model selection. The 70-15-15 split between training, validation, and testing sets was adopted as it is a commonly used strategy in machine learning, particularly for large datasets, as it provides a balanced trade-off between model optimization and unbiased performance evaluation [28].

For completeness, the robustness of the selected partition was further assessed by repeating the analysis using 60-20-20 and 80-10-10 training/validation/test splits. Although the overall performance remained of the same order across the different configurations, measurable differences were observed in the main performance metrics, with relative variations generally below 5% and reaching up to 10% in the least favorable cases. On this basis, the 70-15-15 partition was retained as the most appropriate compromise for the present analysis.

To further refine the dataset before training the model and reduce its dimensionality, a feature selection process was implemented, preserving the input variables that were most informative for predicting the target output. This step is aimed at enhancing model interpretability and generalization while minimizing the risk of overfitting, which is particularly important when dealing with large or high-dimensional datasets, such as the one used in this study [29].

The selection process began with a model-independent filter method called “SelectKBest”, a well-known feature selection technique from the “scikit-learn” library that selects the top *k* features based on univariate statistical tests [30]. In this case, the scoring function used was “mutual_info_classif” [31], a non-parametric measure which estimates the mutual information between each feature and the target variable for classification tasks. Being well-suited to capturing both linear and non-linear relationships, it has proven effective for mixed datasets containing both numerical and categorical variables, with the latter numerically encoded in this study using the pandas function “Categorical”. Specifically, “normal” and “alarm” in the “type” column were coded with “0” and “1”, respectively.

Mutual Information (MI) scores were computed for each pre-processed feature with respect to the binary target variable “type”, and the top 35 features with the highest MI scores were selected. The threshold of 35 was chosen because this subset explained approximately 95% of the total mutual information observed across all input variables, providing a strong balance between model simplicity and predictive power.

Table 2 reports these 35 variables, ranked in descending order of their MI score with respect to the target column (“type”), together with a brief description of their meaning and physical significance.

Following feature selection, an additional decorrelation step was performed to remove redundant features. Using Pearson’s, Spearman’s, and Kendall’s coefficients [32], the correlation between all variables, in groups of two denoted by subscript X and Y, was studied.

The Pearson coefficient $\rho_{XY}$ relates the covariance and the standard deviation of the variables. It is defined as:

(2) $$\rho_{XY}=\frac{\sigma_{XY}}{\sigma_{X}\sigma_{Y}}$$

where $\sigma_{X}$ and $\sigma_{Y}$ are deviations relative to X and Y variables, respectively, and $\sigma_{XY}$ their covariance.

The Spearman coefficient $\theta_{XY}$ is calculated by applying the Pearson formula to the ranked variables R(X) and R(Y), according to the formula reported below:

(3) $$\theta_{XY}=\frac{\sigma_{R(X)R(Y)}}{\sigma_{R(X)}\sigma_{R(Y)}}$$

where $\sigma_{R(X)}$ and $\sigma_{R(Y)}$ represent the standard deviation relative to the ranked variables R(X) and R(Y), and $\sigma_{R(X)R(Y)}$ their covariance.

The Kendall coefficient $\tau_{XY}$ evaluates how similar the ordering of the data is between the two variables and is less sensitive to errors, but particularly effective for small sample sizes. Its formulation is given by:

(4) $$\tau_{XY}=\frac{2(C-D)}{n(n-1)}$$

where C is the number of *concordant* pairs (i.e., pairs where the order of X matches the order of Y), D is the number of *discordant* pairs (i.e., pairs where the order of X and Y do not match), and n is the number of observations.

The above coefficients take values in the range between −1 (perfect negative correlation) and +1 (perfect positive correlation). The value “0” indicates an absence of correlation.

It is worth noting that the correlation analysis was conducted only on the numerical features, as correlation coefficients are not meaningful for variables represented through numerical category codes. A threshold of 0.90 was used to detect potential redundancy. In cases where any pair exceeded this threshold, the feature with the lower mutual information score was to be removed. However, the correlation analysis revealed that none of the selected features exhibited a correlation coefficient above 0.90 under any of the three metrics. Consequently, all 35 features originally selected based on mutual information and reported in Table 2 were retained.

Regarding the input data used for prediction, the vector *x*(*t*) was considered, whose components correspond to the 35 selected columns of the dataset, sampled at a specific time *t*. The machine status prediction type at a future time *t* + *n_s_* + Δ*t* was then generated by analyzing a window of consecutive vectors *n_s_*, i.e., *y*(*t* + *n_s_* + Δ*t*) = *f*([*x*(*t*), *x*(*t* + 1), … *x*(*t* + *n_s_* − 1)]), as schematically represented in Figure 3.

### 2.3. Models Tested and Performance Metrics

A detailed description of each model evaluated for outcome prediction, together with the main hyperparameters (configuration parameters defined before training) available for its tuning [33], is provided below.

**Temporal Convolutional Network** (**TCN**) is a deep learning architecture designed to model sequential dependencies in temporal data [27], replacing traditional recurrent connections with causal and dilated one-dimensional convolutions. This allows it to capture both short- and long-range temporal patterns efficiently without the vanishing gradient issues common in recurrent neural networks. Key hyperparameters include the number of convolutional layers (*n_layers*) and filters per layer (*n_filters*), which determine the network’s representational capacity; the kernel size (*kernel_size*), which controls the temporal receptive field; the dilation rate (*dilation*), which governs the spacing of convolutional filters for each network layer to extend the effective memory of the model; and the dropout rate (*dropout*), which regularizes the network and mitigates overfitting. **Attention mechanism** enables neural networks to dynamically highlight the most informative parts of their input by indicating how much “attention” the model should pay to each element. This improves both the network’s predictive performance and its interpretability.

**k-Nearest Neighbors** (**kNN**) is a non-parametric, instance-based learning algorithm that classifies samples based on the majority class among their *k* closest neighbors in the feature space [34]. Distance is typically computed using metrics such as Euclidean or Manhattan distance. The model’s simplicity and interpretability make it effective for small datasets and low-dimensional spaces, though its performance can degrade with high-dimensional or noisy data. Key hyperparameters include the number of neighbors (*n_neighbors*), which controls the size of the local neighborhood used for prediction and the step interval used for this selection; the weight function (*weights*), which determines whether all neighbors contribute equally or are weighted by distance; and the distance metric (*metric*), which defines how similarity between samples is measured.

**Random Forest** (**RF**) model is an ensemble method that aggregates the predictions of multiple decision trees, each trained on a random subset of the training data and features, a process known as bootstrap aggregation (bagging) [35]. This randomness reduces variance and improves generalization compared to a single decision tree, which is often prone to overfitting. Key hyperparameters include the number of trees (*n_estimators*), which controls ensemble size and stability; the maximum depth of trees (*max_depth*), which regulates model complexity; the minimum number of samples required to split a node (*min_samples_split*) and per leaf (*min_samples_leaf*), both of which influence tree granularity; and the number of features considered at each split (*max_features*), which balances randomness and predictive strength.

**Extremely Randomized Trees** (**Extra Trees**) algorithm follows a similar approach but chooses split thresholds at random rather than optimizing them, which increases diversity among trees [36]. This often leads to faster training and reduced overfitting, though sometimes at the cost of slightly higher bias. Key hyperparameters include the number of learners (*n_estimators*); their maximum depth (*max_depth*); the minimum number of samples required to split a node (*min_samples_split*) and per leaf (*min_samples_leaf*); and the number of features considered at each split (*max_features*).

**Adaptive Boosting** (**AdaBoost**) is a boosting algorithm that builds an ensemble of weak learners, typically shallow decision trees (stumps). Each learner is trained sequentially, with greater weight assigned to previously misclassified samples, forcing subsequent learners to focus on harder cases [37]. This process reduces bias but may increase variance. Its main hyperparameters are the number of weak learners (*n_estimators*); the learning rate (*learning_rate*), which scales the contribution of each learner; and the boosting algorithm (*algorithm*), which determines how weights are updated.

**Histogram-based Gradient Boosting** (**HistGB**) is an ensemble learning method that builds decision trees sequentially, where each new tree is trained to correct the errors (residuals) of the previous ensemble. It minimizes the gradient of a specified loss function, which makes it flexible and powerful for a variety of tasks. In this implementation, continuous features are discretized into histograms (bins), which reduces computational cost and memory usage while maintaining predictive accuracy [38]. Its main hyperparameters include the number of boosting iterations (*max_iter*), which defines the total number of trees added sequentially to the ensemble; the learning rate (*learning_rate*); the maximum depth of trees (*max_depth*); and an L2 regularization term (*l2_regularization*) that helps limit overfitting.

**Extreme Gradient Boosting** (**XGBoost**) is a powerful and regularized implementation of the gradient boosting framework, designed for high scalability [39]. It builds upon the same underlying approach as HistGB, but introduces additional features such as shrinkage, column subsampling, and regularization to combat overfitting. Key hyperparameters include the number of boosting rounds (*n_estimators*); the learning rate (*learning_rate*); the tree complexity (*max_depth*); and the subsampling ratio (*subsample*), which specifies the fraction of training data randomly sampled to grow each tree, helping to reduce overfitting and enhance generalization.

**Light Gradient Boosting Machine** (**LightGBM**) is another gradient boosting framework optimized for speed and memory [40]. It uses a leaf-wise growth strategy instead of level-wise, which allows capturing more complex interactions but increases risk of overfitting if not constrained. Main hyperparameters include the number of trees (*n_estimators*); the learning rate (*learning_rate*); the tree depth (*max_depth*); and the number of leaves (*num_leaves*), which directly controls tree complexity.

**Logistic Regression** (**LR**) is a simple model that estimates the probability of class membership using the sigmoid function [41]. It is interpretable, efficient, and robust for linearly separable data, but limited for non-linear relationships. Its hyperparameters include the regularization strength (*C*), which controls the trade-off between model complexity and overfitting; the penalty type (*penalty*), that helps limit overfitting; and the solver (*solver*), which defines the optimization strategy.

**Ridge Classifier** (**RC**) is a linear model that minimizes squared errors with an L2 penalty [42]. As main hyperparameters, it includes the solver (*solver*); and the regularization parameter (*alpha*), that tunes the penalty strength to prevent overfitting.

**Multi-Layer Perceptron** (**MLP**) is a feed-forward artificial neural network composed of an input layer, one or more hidden layers, and an output layer [43]. Each neuron computes a weighted sum of inputs followed by a non-linear activation function, allowing the model to approximate complex non-linear relationships. In terms of hyperparameters, its architecture is determined by the size of the hidden layers (*hidden_layer_sizes*), that specifies the number and size of hidden layers for each layer; the learning rate (*learning_rate_init*), that controls how weights are updated; and the choice of activation function (*activation*), that strongly influences learning capacity. Although flexible, MLPs may require large datasets and careful tuning to avoid overfitting.

Table 3 summarizes the tuned hyperparameters and the corresponding search ranges considered for each model [44,45], in accordance with the tuning procedure outlined in Section 2.2.

In addition, to address the class imbalance between the two possible machine status outcomes (“normal” and “alarm”), the parameter *class_weight = “balanced”* was applied in all classification models. Specifically, the balancing is a procedure that automatically adjusts the weight of each class inversely proportional to its frequency in the training data, thereby mitigating bias toward the majority class. This ensures that misclassifications of the minority class are penalized more heavily during training, improving the model’s ability to detect alarm states without compromising overall performance.

The comparison among the models was based on the F_1_ score, which was adopted as the reference metric for the overall evaluation of model performance. Additional complementary metrics were also considered to provide a more comprehensive characterization of classifier behavior: recall (or sensitivity or True Positive Rate (TPR)) to assess the ability to correctly detect alarm events, specificity and False Alarms per treatment hour (FA/h) to quantify incorrect alarm generation under normal operating conditions, and the Area Under the Receiver Operating Characteristic Curve (AUROC) and the Area Under the Precision-Recall Curve (AUPRC) to evaluate the discriminative ability of the models across different classification thresholds.

Since several of the performance metrics considered in this study are derived from the classification outcomes, it is first useful to introduce the confusion matrix [46], which summarizes correctly and incorrectly classified samples according to their actual and predicted classes along the primary and the secondary diagonal, respectively. In particular, True Positives (TP) correspond to alarm-related samples correctly classified as positive, whereas True Negatives (TN) represent normal samples correctly classified as negative. Conversely, False Positives (FP) are normal samples incorrectly classified as alarms, while False Negatives (FN) are alarm-related samples incorrectly classified as normal.

Based on these quantities, F_1_ score is defined as:

(5) $$F1\;\mathrm{score}\;=2\cdot \frac{\mathrm{Precision}\cdot \mathrm{Recall}}{\mathrm{Precision}+\mathrm{Recall}}$$

where in turn Precision and Recall are defined, respectively, as:

(6) $$\mathrm{Precision}=\frac{\mathrm{True}\;\mathrm{positives}}{\mathrm{True}\;\mathrm{positives}+\mathrm{False}\;\mathrm{positives}}$$

(7) $$\mathrm{Recall}=\frac{\mathrm{True}\;\mathrm{positives}}{\mathrm{True}\;\mathrm{positives}+F\mathrm{alse}\;\mathrm{negatives}}$$

Specifically, F_1_ score is a useful performance metric when class imbalances are present in the dataset [47]. Its value varies between 0 and 1, indicating poor and excellent performance, respectively.

Precision expresses the fraction of samples predicted as alarms that are actually associated with an alarm event, whereas recall quantifies the fraction of actual alarm-related samples that are correctly detected by the classifier. The latter is particularly relevant in the present study, since alarm-related samples represent the minority class [46]. It ranges between 0 and 1, with higher values indicating a lower occurrence of false negatives.

In addition, specificity was considered to complement recall by evaluating the ability of the classifier to correctly recognize negative instances [46]. In the present context, it therefore measures the capability of the model to correctly identify operating conditions not associated with an impending alarm. Specificity ranges between 0 and 1, with higher values indicating fewer false-positive predictions, and is defined as:

(8) $$\mathrm{Specificity}=\frac{\mathrm{True}\;\mathrm{negatives}}{\mathrm{True}\;\mathrm{negatives}+F\mathrm{alse}\;\mathrm{positives}}$$

While F_1_ score, recall, precision, and specificity are computed for a given classification threshold, AUROC provides a threshold-independent measure of the ability of the classifier to discriminate between positive and negative instances, describing the relationship between the TPR and the False Positive Rate (FPR) [48], that is the complementary metric of specificity. AUROC ranges between 0 and 1, with a value of 0.5 corresponding to discrimination equivalent to random classification and values approaching 1 indicating increasingly effective discrimination.

The AUPRC instead summarizes the trade-off between precision and recall across different classification thresholds. This metric is particularly informative for imbalanced classification problems, as it focuses directly on the performance achieved on the positive class [48]. Higher AUPRC values indicate a greater ability of the classifier to detect positive instances while limiting false-positive predictions.

Finally, FA/h were considered as an application-oriented metric to quantify the practical burden associated with incorrect alarm predictions during treatment. Unlike sample-based metrics such as specificity, this metric accounts for distinct false-alarm episodes and normalizes their occurrence by the total treatment duration, providing a more intuitive indication of how frequently unnecessary alarms may be generated under real operating conditions [49]. A false-alarm episode was defined as a continuous sequence of consecutive false-positive predictions within the same treatment session, so that consecutive false-positive samples were counted as a single event. The metric was defined as:

(9) $$FA/h\;=\frac{\mathrm{Number}\;\mathrm{of}\;\mathrm{false}\;\mathrm{alarms}\;\mathrm{episodes}}{\mathrm{Total}\;\mathrm{treatment}\;\mathrm{duration}}$$

where a value of zero false alarms per treatment hour represents the ideal condition of absence of erroneous alarm episodes during the treatment.

For each parameter and configuration, the standard deviation was also computed to account for variability across cross-validation folds, and based on the overall performance obtained from this comparative analysis, the best-performing model was then selected for further evaluation and analysis.

## 3. Results

This section presents the results obtained in this study. Specifically, it is organized into two main parts: the first compares the performance of all the tested models to identify the most accurate one; the second one analyzes the sensitivity of the selected model to variations in the main temporal parameters, providing a comprehensive evaluation of its predictive robustness.

### 3.1. Performance Comparison

Table 4 reports the hyperparameter settings of the best-performing configuration identified for each machine learning algorithm through 5-fold cross-validation, together with the fixed configuration adopted for the deep learning model.

Table 5 summarizes the predictive performance of each best configuration in terms of F_1_ score, precision, recall, specificity, AUROC, AUPRC, and FA/h, with each metric reported as mean ± standard deviation.

Specifically, these results correspond to an initial setting with a time window of Δ*t* = 20 rows and *n_s_* = 10 rows, adopted as a baseline configuration to be later expanded once the optimal model among the tested settings had been identified. The value *n_s_* = 10 rows was chosen as it represents the minimum sequence length required to capture sufficient historical context, across a diverse set of clinical and machine-derived inputs, and to provide the model with a coherent and informative representation of the patient–machine interaction prior to prediction. In parallel, the time window Δ*t* = 20 rows represents a reasonable lower bound, as it ensures a minimal interval for effective intervention before the alarm is triggered. As a consequence, it was chosen as the critical threshold below which the opportunity to respond to early warning signs becomes limited. Tested on the 15% of the data held out as a hold-out validation set, the DL model and the best configuration for each ML model identified during hyperparameter tuning yielded the performance metrics reported in Table 6.

Here, the models achieved results consistent with those observed in cross-validation, reinforcing the robustness of the comparative analysis, with Random Forest emerging as the most reliable approach. It is worth noting that the Extremely Randomized Trees algorithm also achieved competitive results, both in 5-fold cross-validation (Table 5) and in the validation phase (Table 6). However, a limitation of the Extra Trees model emerged in terms of recall. Specifically, Extra Trees achieved an average recall of approximately 0.93 across the 5-fold cross-validation and ~0.92 in the validation phase, compared with ~0.97 and ~0.95, respectively, for Random Forest. A similar, although less pronounced, difference was observed in terms of precision, with Extra Trees reaching ~0.98 across the 5-fold cross-validation and ~0.97 in the validation phase, while Random Forest achieved values close to 0.99 and 0.98, respectively. Consequently, Extra Trees showed a larger imbalance between precision and recall, indicating a greater tendency to miss positive alarm samples despite maintaining a low number of false positive predictions.

This difference was also reflected in the overall classification performance. Random Forest achieved a higher F_1_ score (~0.98) than Extra Trees (~0.95), and although both approaches showed nearly perfect discrimination in terms of AUROC (~0.99 for Random Forest and ~0.98 for Extra Trees), Random Forest also provided a slightly higher AUPRC (~0.98 vs. ~0.96), which is particularly relevant given the marked class imbalance characterizing the dataset.

Importantly, the superiority of Random Forest was also evident from an operational perspective, with an average of 0.73 FA/h for Random Forest compared with 0.91 FA/h for Extra Trees. Specifically, lower FA/h indicates a reduced burden of unnecessary warnings during treatment, which is particularly relevant for the practical deployment of an alarm-prediction system.

Overall, Random Forest therefore provided the most favorable compromise between alarm recall, precision, class-imbalance-aware performance, and false-alarm burden. Given the importance of correctly anticipating true alarms while simultaneously limiting unnecessary warnings, it was adopted as the reference model for the subsequent analyses presented in the following section.

### 3.2. Parametric Sensitivity Assessment

The results reported in Table 5 and Table 6 show that the Random Forest classifier achieved the best performance, combining high predictive accuracy with consistent results across hyperparameter settings. Consequently, it was used to perform a parametric study aimed at assessing the sensitivity of its best-performing configuration to variations in key input Δ*t* (and Δ*t_s_*, equal to Δ*t* expressed in seconds) and *n_s_*.

The detailed values of all performance metrics derived from the sensitivity analysis of the Random Forest classifier, obtained using the remaining 15% of the dataset not used in any previous stage, are shown in Table 7.

Specifically, Table 7 shows the trend of F_1_ score, precision, recall, specificity, AUROC, AUPRC, and FA/h obtained by the model as a function of Δ*t* (and Δ*t_s_*) for each *n_s_* considered. Values of *n_s_* equal to 10, 20, 30 and 40 rows, and Δ*t* of 20, 50, 80, 120 and 150 rows (corresponding to Δ*t_s_* equal to 10, 25, 40, 60, and 75 s, respectively), were taken into account.

In terms of overall predictive performance, increasing the sequence length *n_s_* did not provide a consistent improvement across the evaluated classification metrics, although longer sequences generally resulted in a modest reduction in FA/h. A clearer trend was observed with increasing prediction horizon Δ*t*, which was generally associated with a progressive deterioration in F_1_ score, precision, recall, and specificity, together with lower AUROC and AUPRC values. At the same time, FA/h consistently increased as the prediction horizon was extended, reflecting the greater difficulty of generating reliable warnings further in advance.

Based on these results, the configuration with a prediction horizon of Δ*t* = 120 rows and a sequence length of *n_s_* = 10 rows was identified as providing the most favorable trade-off between prediction anticipation and classification performance. Specifically, this configuration allows alarms to be anticipated potentially up to one minute in advance while maintaining satisfactory predictive performance, with an F_1_ score of approximately 0.85, together with a specificity of ~0.99, an AUROC of ~0.99, and an AUPRC of ~0.94.

Although shorter prediction horizons yielded higher classification performance, the additional anticipation provided by the 60 s horizon was considered to outweigh the moderate loss in predictive accuracy. Furthermore, the relatively short sequence length (*n_s_* = 10) limits the computational burden, supporting the suitability of this configuration for real-time implementation.

For this specific scenario, the confusion matrix is represented in Figure 4.

For the test dataset, 15,607 true positives were identified out of 17,856 alarms, with 3173 false positives and 2249 false negatives.

Although direct comparisons should be interpreted with caution because of differences in alarm definitions, datasets, prediction targets, sampling frequencies, and evaluation protocols, these results can be contextualized with respect to related applications in hemodialysis. For instance, Wang and Xu [50], using a Random Forest model to predict hemodialysis alarm events, reported a recall of 72.30% and a precision of 68.90%, compared with the recall of ~87% and precision of ~83% obtained here for the 60 s prediction horizon. Similarly, Xie et al. [18] reported a precision of 89.20% and a recall of 81.30% for Convolutional Neural Network (CNN)-based recognition of dialysis-machine alarms, although their approach addressed alarm recognition rather than advance prediction.

In the related context of real-time prediction of intradialytic hypotension, Zhang et al. [51] reported that a false-positive rate of 10% was considered clinically and operationally acceptable by healthcare professionals, corresponding to a recall of 65% and a specificity of 90%. For comparison, the present model achieved a sample-level false-positive rate of 0.86% at the 60 s prediction horizon, corresponding to a relatively limited false-positive burden at the sample level.

However, since the dataset is sampled every 0.5 s, unlike the approximately 30 min intervals in Zhang et al. [51], sample-level metrics do not directly reflect distinct alarm occurrences. Indeed, an alarm episode may include multiple consecutive alarm-labeled samples, while consecutive false-positive predictions may correspond to a single false warning from an operational perspective. Therefore, to provide a more clinically and operationally meaningful assessment of the selected configuration (Δ*t* = 120 rows and *n_s_* = 10 rows), an additional event-level analysis was performed. Specifically, the proportion of distinct alarm episodes successfully anticipated, and the distribution of prediction lead times were evaluated. The corresponding results are reported in Table S2 of the Supplementary Materials.

## 4. Discussion

In this section, the results presented previously are discussed in order to provide an interpretation and to analyze their main implications.

First, the comparative performance of the tested models presented in Section 3.1 is examined, with particular emphasis on advantages and disadvantages of each model. Subsequently, the effects of the main temporal parameters on predictive performance are analyzed, considering the configuration presented in Section 3.2.

### 4.1. Model Performance Analysis

As shown in Section 3.1, Table 5 and Table 6 report the performance of the best configuration for each model tested. Among the tree-based ensemble methods, Random Forest and Extremely Randomized Trees achieved the highest F_1_ score, but the greater importance of recall over precision for this task, together with the more favorable FA/h values, led to the selection of Random Forest as the best model. Specifically, although in medicine both false positives and false negatives are clinically relevant, as the former can lead to unnecessary actions and the latter may result in missed event, in binary classification studies, recall is typically prioritized over precision. A higher recall represents a more conservative approach that reduces the likelihood of missing positive cases, especially in real-world operation, where rare but critical adverse events must be detected reliably despite their low frequency.

Regarding neural models, approaches such as the Multi-Layer Perceptron and the Temporal Convolutional Network with Attention exhibited consistent yet inferior results. Specifically, although the TCN architecture is designed to capture temporal dependencies and the attention mechanisms to focus on relevant temporal patterns, their performance was likely constrained by the pronounced class imbalance and the limited number of alarm samples [52]. In such conditions, deep architectures tend to bias toward the majority class, which could explain the performance gap observed with respect to ensemble-based approaches. Moreover, the increased network complexity of the TCN and MLP models, characterized by a large number of trainable parameters, may have induced overfitting, preventing the networks from generalizing on unseen samples.

k-Nearest Neighbors and boosting-based models, such as Adaptive Boosting, Histogram-based Gradient Boosting, Extreme Gradient Boosting, and Light Gradient Boosting Machine showed solid but comparatively lower performance, which may be partly attributable to their more limited ability to handle highly imbalanced datasets [53]. Finally, models such as Logistic Regression and the Ridge Classifier achieved comparatively lower performance across the evaluated metrics, reflecting their limited capacity to model complex, non-linear relationships within the patient–machine interaction data [54].

However, when considered as a whole, the concordance observed among the independent dataset partitions suggests that the models did not overfit to specific folds but rather captured reproducible patterns. This stability across cross-validation folds represents a strong indicator of model consistency and further supports the robustness of the comparative analysis.

### 4.2. Effect of Temporal Parameters on Model Performance

The parametric study shown in Table 7 illustrates an overall decrease in performance as Δ*t* increases, while increasing *n_s_* did not provide a consistent performance benefit.

At first sight, increasing the sequence length should allow the model to capture temporal dynamics of the machine status and thus enhance prediction. However, this effect is not observed, most likely because the most informative data lies in the most recent samples, while more distant observations contribute little to the prediction. Moreover, longer input sequences introduce a higher number of features, which increases the dimensionality of the problem and makes the classification task more complex. At the same time, extending the input window often incorporates redundant or noisy samples. This cumulative noise dilutes the relevant precursors of the alarm event, ultimately reducing or keeping constant the discriminative power of the model.

On the contrary, the decrease in model performance as Δ*t* increases is intuitive since anticipating alarms further in advance introduces greater uncertainty, with the current system state becoming less directly connected to the future alarm. As a result, predictive patterns weaken, and both precision and recall decline.

Specifically, the reduction in most performance metrics appears to be gradual for shorter predictive horizons, but becomes much more pronounced beyond Δ*t* = 120 rows, consistently across all values of *n_s_* investigated. This behavior suggests that, up to a certain horizon, the system state still retains a sufficient connection with the upcoming alarm, allowing the model to extract useful predictive cues. Once Δ*t* exceeds this threshold, however, the temporal distance between input features and the alarm event becomes too large, and the predictive signal rapidly deteriorates. Consequently, the model’s discriminative ability drops sharply, leading to a steeper decline in performance observed beyond Δ*t* = 120 rows.

Based on these considerations, the configuration having Δ*t* = 120 rows and *n_s_* = 10 rows was identified as the most balanced, as shown by the parametric study presented in Section 3.2.

### 4.3. Limitations of the Work

Despite the promising results, this study presents some limitations that should be acknowledged.

First, the dataset was collected from a single type of hemodialysis machine and includes a limited number of treatment sessions. Although the dataset is large in terms of temporal resolution and covers a substantial monitoring period, this constraint may limit the generalizability of the findings to other devices, clinical settings, or patient populations.

Second, the predictive task focused exclusively on the binary detection of alarm occurrence, without distinguishing among different alarm types. While this choice simplifies the problem and is clinically relevant for early intervention, it does not exploit the full informational content of the available alarm codes, which could provide more targeted and actionable predictions.

Moreover, the strong class imbalance inherent to alarm prediction represents an intrinsic challenge. Although class weighting strategies were applied and recall-oriented evaluation criteria were adopted, rare alarm events remain difficult to model, particularly for longer prediction horizons. This limitation was reflected in the progressive performance degradation observed as Δ*t* increased.

Finally, the selected Random Forest model treats past observations as independent feature vectors, without explicitly modeling temporal dependencies. While this approach proved effective for short- and medium-term prediction, it may limit performance for longer anticipation times, suggesting that more advanced architectures could further improve long-range predictive capability.

## 5. Conclusions

In this study, an AI-based predictive tool for early alarm detection in dialysis treatments was developed, with a focus on anticipating machine-generated alarms that may reflect patient- or process-related complications. By exploiting data collected from hemodialysis equipment and applying a Random Forest classifier, the proposed model achieved high predictive performance (F_1_ score of 0.85 for Δ*t*_s_ = 60 s) and proved capable of providing clinicians with valuable anticipation time before alarms were triggered.

The results showed that short predictive windows combined with relatively small input sequences offered the most effective compromise between accuracy and robustness. In particular, a configuration with Δ*t* = 120 rows and *n_s_* = 10 rows allowed the model to predict alarms up to one minute (Δ*t*_s_ = 60 s) in advance, achieving good overall performance and showing promising potential for future clinical application, although prospective validation would be required to assess its impact on alarm burden and patient safety.

Nevertheless, the lack of a clear improvement in performance for larger *n_s_* values and the degradation for larger Δ*t* values reflect, respectively, the time-independent nature of tree-based models and the progressive weakening of the correlation between inputs and outputs as the temporal gap increases.

Furthermore, the results demonstrated that conventional machine learning architectures remain, for the time being, more computationally efficient than deep learning architectures for well-defined classification tasks based on structured data. This advantage stems primarily from their ability to effectively capture interactions among variables, while requiring fewer data and computational resources than are generally needed to fully exploit the potential of deep neural networks.

However, future work will investigate more sophisticated and carefully optimized DL models beyond the TCN evaluated in this study, with the aim of determining whether they can outperform machine learning for this specific task, while extending the predictive horizon and maintaining high accuracy.

Additionally, efforts will be directed toward enabling the identification of the specific type of alarm, in addition to its occurrence, as this represents another variable (“code” column) available in the dataset.

Further studies should assess the scalability of the model across hardware and clinical settings, as well as its performance on larger, external, and multi-center datasets, to ensure robustness and practical applicability. In particular, although the computational burden appears compatible with the real-time deployment, the relatively limited number of treatment sessions and devices considered in the present study may not fully capture the variability associated with different dialysis machine models, patient populations, and clinical centers. Future validation should therefore involve larger and more heterogeneous datasets collected across multiple devices and institutions to evaluate the generalizability of the model under diverse operating and clinical conditions.

All together, these advances could drive the integration of the proposed system into existing clinical workflows and the evolution toward smarter dialysis systems, where AI-based predictive tools may support earlier identification of impending alarm conditions, reduce unplanned interruptions, and improve the overall reliability of treatment delivery.

## Figures and Tables

**Figure 1:**
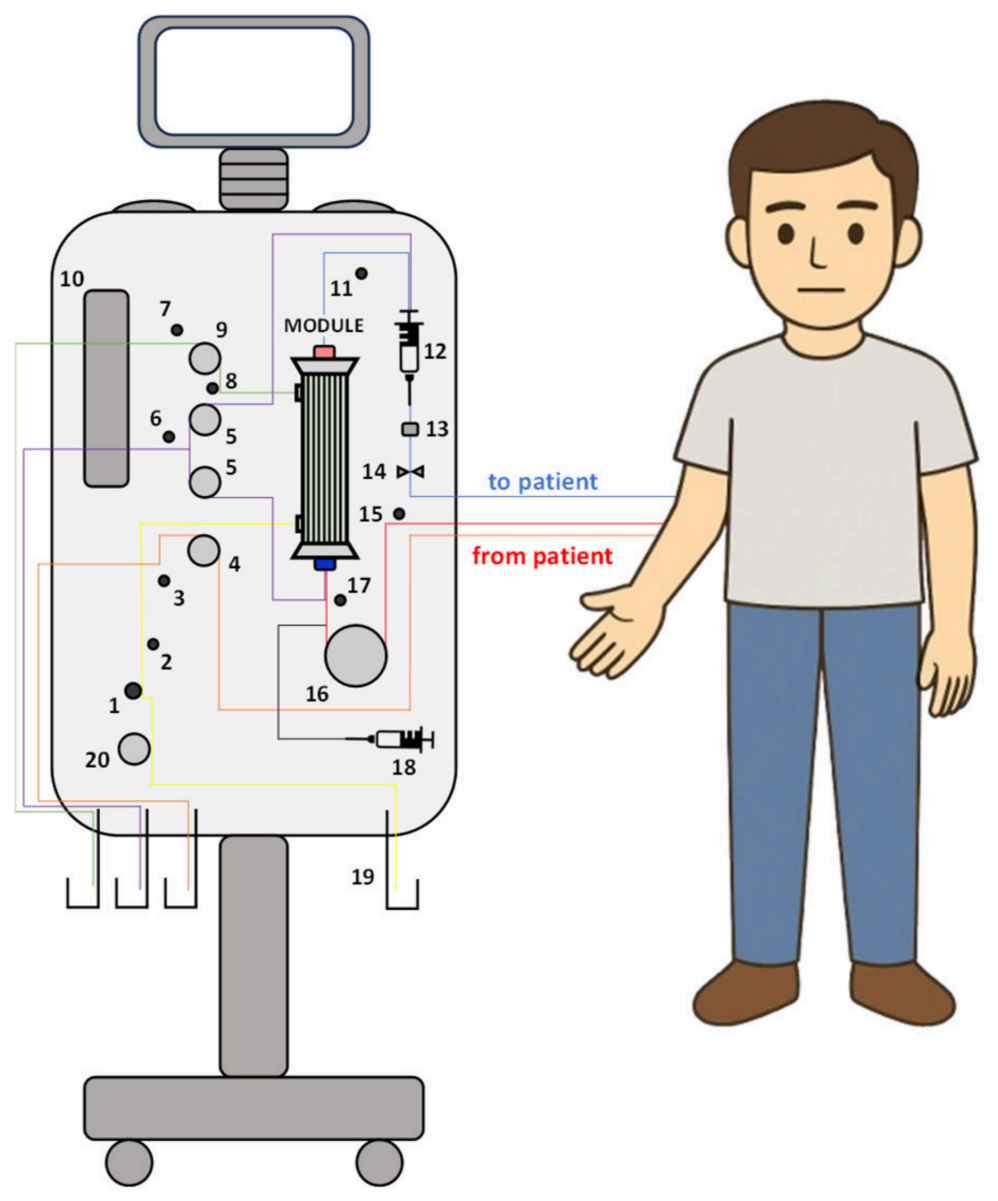
Schematic of the hemodialysis circuit, showing the main components of the extracorporeal blood and dialysate circuits. The numbered elements indicate: (1) blood leakage detector sensor; (2) ultrafiltrate pressure sensor; (3) pre-blood pressure sensor; (4) pre-blood pump; (5) substitution pumps (pre- and post-filter); (6) substitution pressure sensor; (7–8) dialysate pressure sensors (pre- and post-pump); (9) dialysate pump; (10) dialysate and substitution fluid heater; (11) return pressure sensor; (12) air removal chamber; (13) air pressure sensor in the blood line; (14) safety clamp on the return line; (15) access pressure sensor; (16) blood pump; (17) pre-filter pressure sensor; (18) anticoagulant syringe pump; (19) load cells for dialysate, substitution, and ultrafiltrate bags; (20) ultrafiltrate pump.

**Figure 2:**
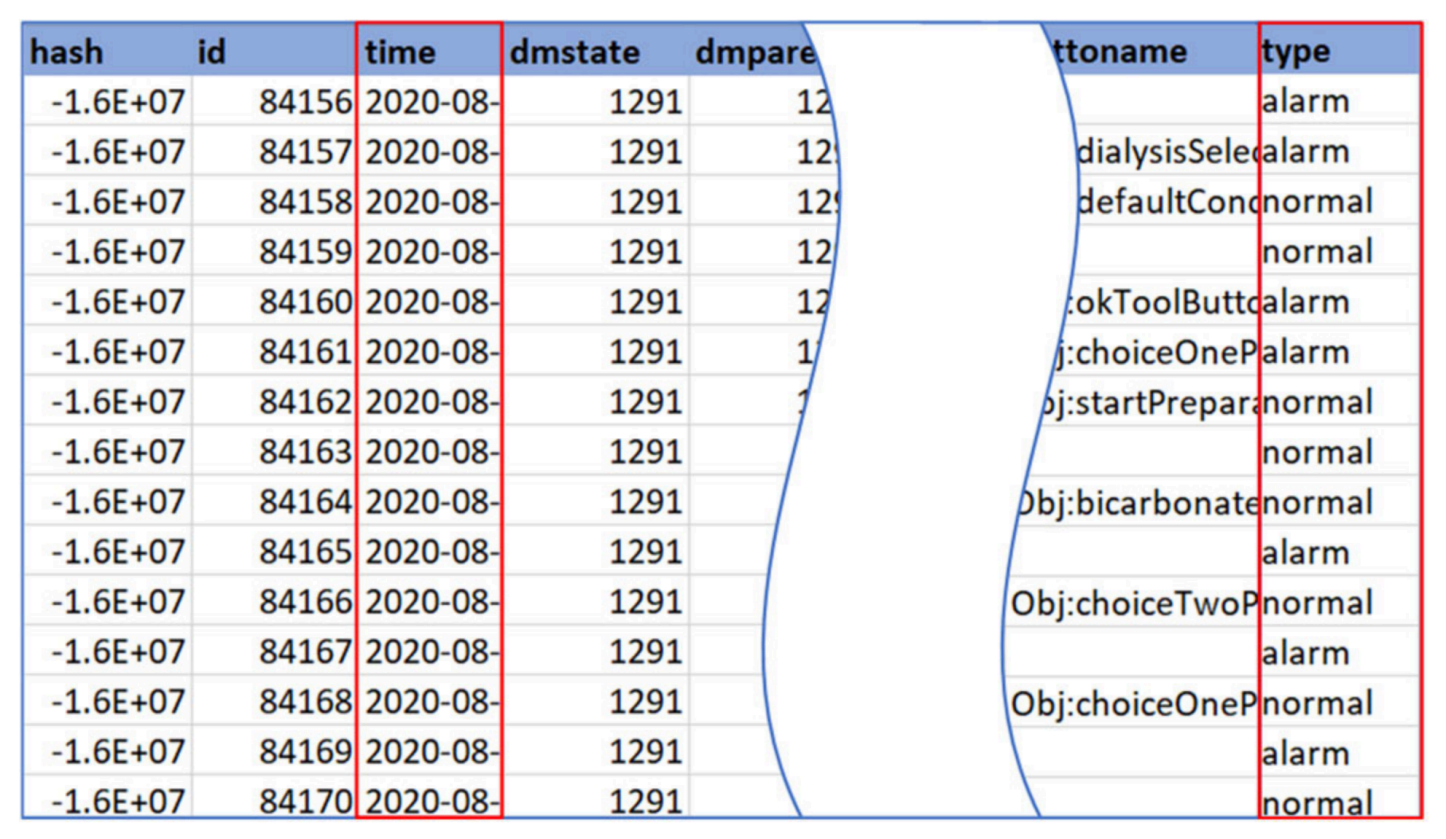
Two portions of the 93 columns from the model training file. On the left, first columns of the training file: the red box surrounds the “dmstate” column, identifying the treatment phase. On the right, last columns of the training file: in the red box, the column “type” identifies the machine’s binary status (alarm/normal). Each row in the dataset corresponds to a measurement taken every 0.5 s.

**Figure 3:**
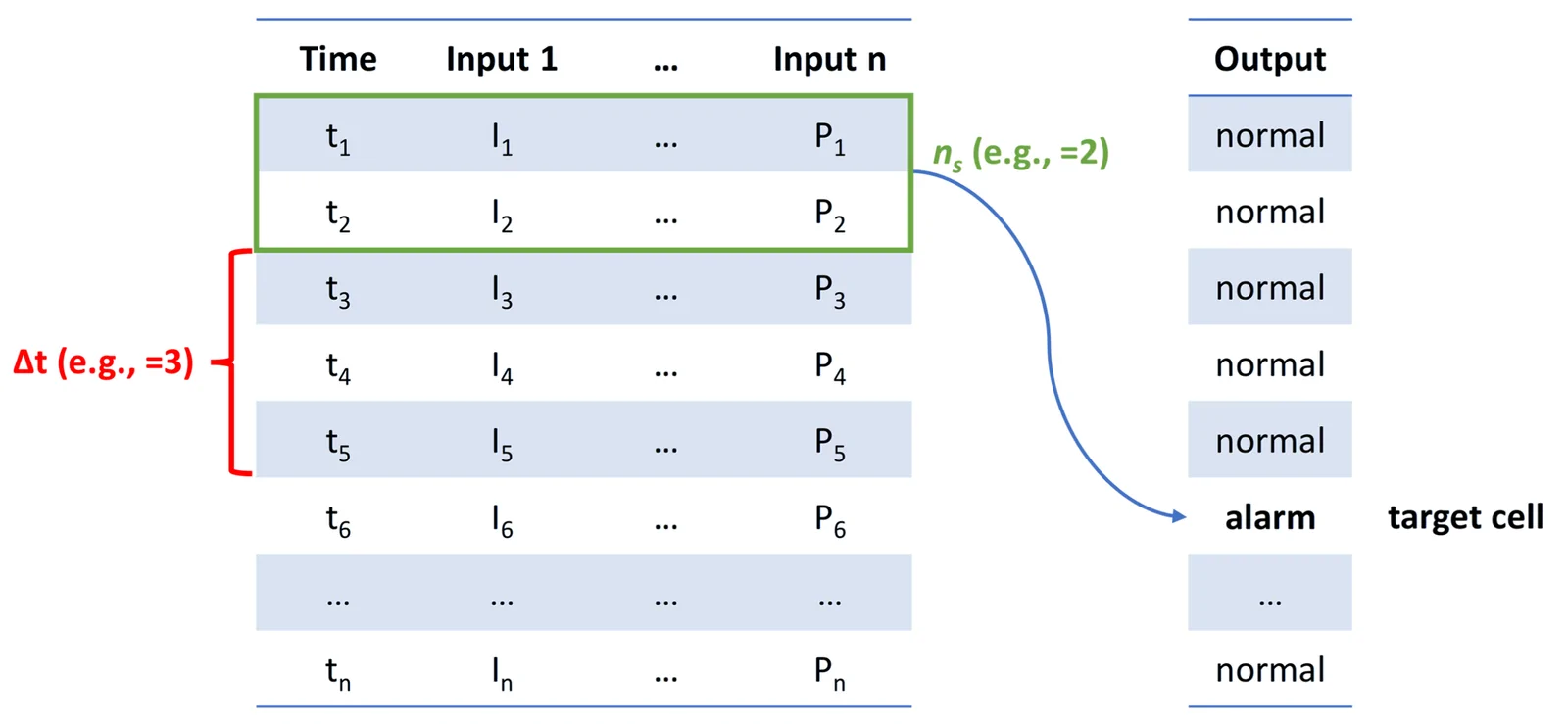
Graphical representation of the prediction model. The number of steps *n_s_* is the number of rows known at the time the model makes the prediction (*n_s_* = 2 rows in the figure). The predictive time window Δ*t* is the number of rows between the current and future condition to be predicted (Δ*t* = 3 rows in the figure). Each row in the dataset corresponds to a measurement taken every 0.5 s.

**Figure 4:**
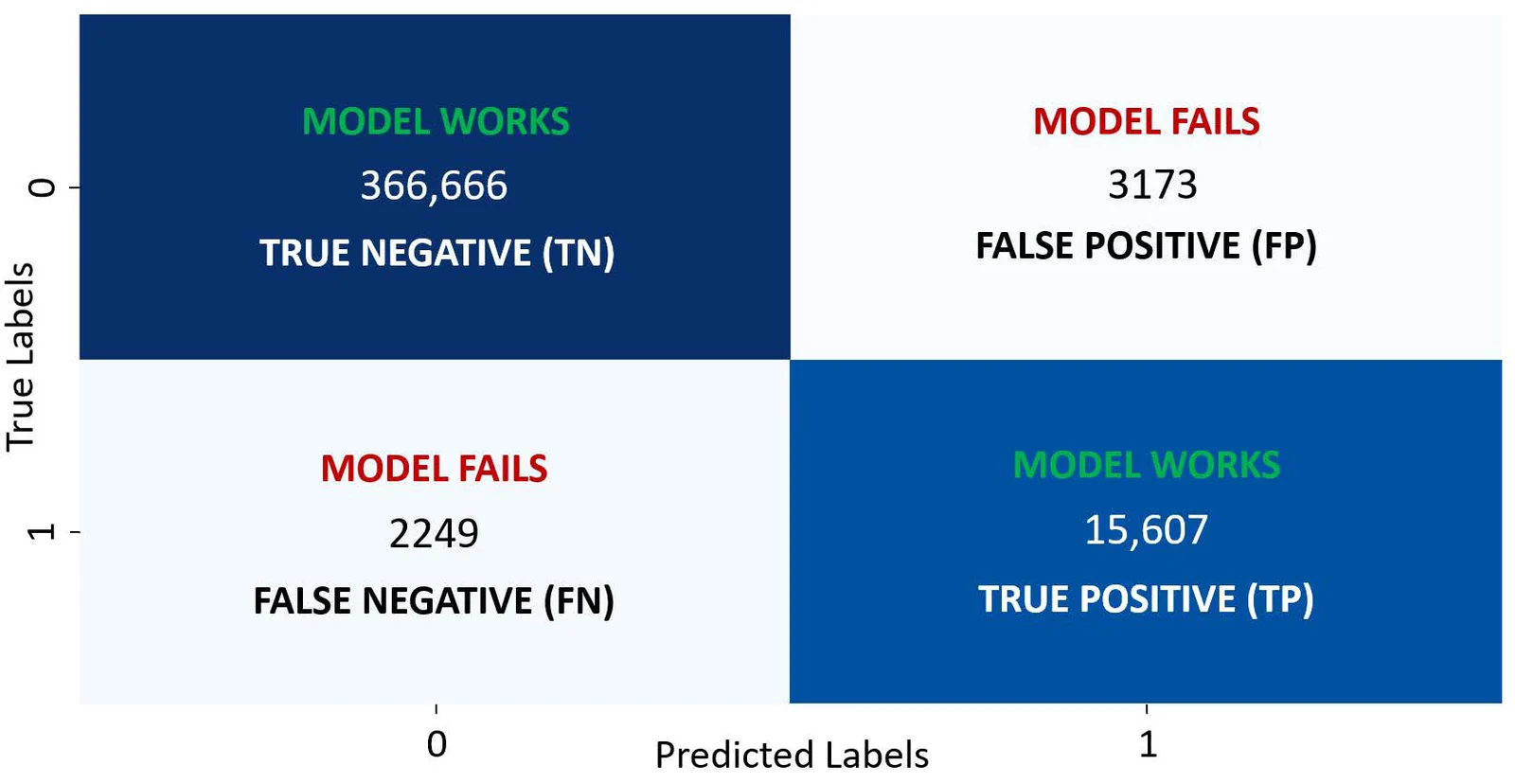
Confusion matrix obtained with Δ*t* = 120 rows (where Δ*t* is the number of rows between the current and future condition to be predicted) and *n_s_* = 10 rows (where *n_s_* is the number of rows known at the time the model makes the prediction). The matrix refers to 15% of the total dataset used as the test set, and each row of the analysis corresponds to a measurement taken every 0.5 s.

**Table 1:** Patient- and process-related monitoring systems and the corresponding alarm conditions representing the positive class (“type” = alarm) in the dataset.

Control System	Control Objective
Vascular access pressure monitoring system	Abnormal pressures in the arterial or venous line, or across the dialyzer membrane, suggesting impaired blood flow to/from the patient.
Blood circuit pressure control system	Disconnection or leakage in the line connecting the patient’s vascular access to the circuit, exposing the patient to risk of blood loss or air entry.
Blood flow and pump control system	Interruption of blood flow returning to the patient due to clamp activation or pump stop, which may lead to inadequate perfusion or hemolysis.
Air/foam detection and alarm system	Detection of air and foam in the venous blood line returning to the patient, indicating a potential air embolism risk or blockage preventing safe blood reinfusion.
Anticoagulation monitoring system	Inadequate or interrupted heparin delivery during treatment, increasing the risk of clot formation in the extracorporeal circuit and subsequent loss of blood volume for the patient.
Ultrafiltration control system	Deviations from the prescribed fluid removal target, possibly leading to incorrect patient fluid balance or intradialytic hypotension.
Blood pressure measurement system	Failure or interruption of automatic blood pressure measurement during therapy, resulting in loss of real-time information on the patient’s hemodynamic stability.
Self-testing and timer control system	Temporary interruption due to automatic safety or verification cycles that delay blood return or alter patient’s flow rates.

**Table 2:** List of the 35 features selected through the Mutual Information (MI)-based feature selection using the SelectKBest method. Variables are ranked according to their MI with respect to the target column (“type”), and each description summarizes their technical or physical meaning.

Variable Name	Definition
Parent state of the dialysate side	Identifier of the higher-level operational state (e.g., preparation, treatment, rinse) of the dialysate side in the system state machine.
Child state of the dialysate side	Identifier of the specific operational substate of the dialysate side in the system state machine.
Post-arterial pump pressure	Measurement of the blood pressure downstream of the arterial pump, before the dialyzer (mmHg).
Pre-arterial pump pressure	Measurement of the suction pressure in the blood inlet line (mmHg).
Delivery pump actuation signal	Measurement of the digital control signal (bit) that activates the dialysate delivery pump.
Venous line pressure	Measurement of the blood pressure on the venous return side, downstream of the dialyzer (mmHg).
Foam detection signal	Measurement of the signal indicating the presence of foam in the venous blood line (bit).
Transmembrane pressure	Measurement of the difference between the pressure on the blood side and that on the dialysate side through the dialyzer membrane (mmHg).
Delivery flow rate	Measurement of the actual dialysate flow rate delivered to the dialyzer (mL/min).
General blood pressure	Measurement of the general blood circuit pressure (mmHg).
Delivery pump flow rate	Measurement of the flow rate delivered by the dialysate delivery pump (mL/min).
Ultrafiltration flow rate	Measurement of the actual ultrafiltration flow rate (g/min).
Current patient weight loss	Measurement of the estimated weight of fluid removed during treatment (g).
Arterial pump revolutions	Measurement of the revolutions of the arterial pump encoder (revolutions).
Ultrafiltration pump actuation signal	Measurement of the control signal that activates the ultrafiltration pump (bit).
Dialysate output conductivity (filtered)	Measurement of the dialysate conductivity at the dialyzer outlet after digital filtration (mS/cm).
Dialysate output conductivity (not filtered)	Measurement of the raw conductivity value before digital filtering (mS/cm).
Dialysate outflow pressure	Measurement of the dialysate pressure at the dialyzer outlet (mmHg).
Ultrafiltration pump flow rate	Measurement of the volume delivered by the ultrafiltration pump (g/min).
Total conductivity	Measurement of the conductivity of the dialysate mixture (mS/cm), sum of all the preparation phases.
Infusion pump revolutions	Measurement of the infusion pump encoder count to control the reinfusion volume (revolutions).
Venous pump revolutions	Measurement of the revolutions of the venous pump encoder (revolutions).
Parent state of the blood side	Identifier of the main phase of blood circuit operation in the system state machine.
Child state of the blood side	Identifier of the substate of the blood side (e.g., priming, rinse-back) in the system state machine.
Total conductivity (filtered)	Measurement of the processed conductivity of the dialysate mixture (mS/cm), sum of all the preparation phases.
Venous pressure	Measurement of the pressure in the venous line (mmHg).
Pre-filter pressure	Measurement of the pressure before the dialyzer filter on the dialysate side (mmHg).
Second-step conductivity	Measurement of the conductivity in the second mixing step of the dialysate (mS/cm).
Tank temperature	Measurement of the main water tank temperature (°C).
Speed of the second-step conductivity pump	Measurement of the rotation speed of the second-stage mixing pump (revolutions per minute).
Ultrafiltration circuit pressure	Measurement of the pressure inside the ultrafiltration control circuit (mmHg).
Speed of the first-step conductivity pump	Measurement of the rotation speed of the first-stage mixing pump (revolutions per minute).
Pre-dialyzer blood temperature	Measurement of the blood temperature before entering the dialyzer (°C).
Pre-dialyzer dialysate temperature	Measurement of the dialysate temperature before entering the dialyzer (°C).
Air detection signal	Measurement of the signal indicating the presence of air in the blood circuit (bit).

**Table 3:** Summary of the compared models, tuned hyperparameters, and corresponding search ranges. For each model, ten random configurations were evaluated from all possible combinations. Model acronyms are listed in the Abbreviations section.

Model Tested	Hyperparameters Tuned + Range of Values Considered for Each Model Configuration
TCN + Attention	n_layers: [3]; n_filters: {(64, 64, 128)}; kernel_size: [3]; dilation: {[1, 2, 4]}; dropout: [0.15]
kNN	n_neighbors: [3:2:31]; weights: {uniform, distance}; metric: {euclidean, manhattan}
RF	n_estimators: [50, 400]; max_depth: [3, 40] + none; min_samples_split: [2, 10]; min_samples_leaf: [1, 5]; max_features: {sqrt, log2}
Extra Trees	n_estimators: [50, 400]; max_depth: [3, 40] + none; min_samples_split: [2, 10]; min_samples_leaf: [1, 5]; max_features: {sqrt, log2}
AdaBoost	n_estimators: [50, 400]; learning_rate: [0.01, 1]; algorithm: {SAMME, SAMME.R}
HistGB	max_iter: [100, 300]; learning_rate: [0.01, 1]; max_depth: [3, 40] + none; l2_regularization: [0, 0.2]
XGBoost	n_estimators: [50, 400]; learning_rate: [0.01, 1]; max_depth: [3, 40]; subsample: [0.6, 1]
LightGBM	n_estimators: [50, 400]; learning_rate: [0.01, 1]; max_depth: [3, 40] + none; num_leaves: [30, 260]
LR	C: [0.01, 10]; penalty: {l2, none}; solver: {lbfgs, saga}
RC	alpha: [0.1, 20]; solver: {auto, lsqr, sparse_cg}
MLP	hidden_layer_sizes: {(50), (100), (50,50), (100,50)}; activation: {relu, tanh}; learning_rate_init: [0.0005, 0.0200]

where square brackets [*a*, *b*] denote a continuous range from which values are randomly sampled, the notation [*a*:*s*:*b*] denotes the integer values from *a* to *b* with step *s* (e.g., [3:2:31] corresponds to 3, 5, 7, …, 31), curly brackets {…} denote discrete sets of options, ‘uniform’ refers to equal weighting of all neighbors in k-Nearest Neighbors, while ‘distance’ assigns weights inversely proportional to distance, ‘euclidean’ refers to the straight-line distance between points in the feature space, ‘manhattan’ denotes the distance as the sum of absolute differences along each feature dimension, ‘sqrt’ represents the square root of the number of features considered at each split, ‘log2’ corresponds to the base-2 logarithm of the number of features used at each split, ‘SAMME’ denotes an AdaBoost extension for multi-class classification that relies on discrete class predictions for weight updating, ‘SAMME.R’ represents a SAMME variant that employs class probability estimates for the same purpose, ‘lbfgs’ is a quasi-Newton optimization algorithm efficient for small to medium-sized datasets, ‘saga’ refers to an optimization algorithm suitable for large and sparse datasets, ‘l2’ indicates the L2 regularization applied to penalize large coefficients, ‘auto’ automatically selects the most appropriate solver based on the data characteristics, ‘lsqr’ denotes an iterative least-squares solver, ‘sparse_cg’ refers to a conjugate gradient solver optimized for sparse data, ‘relu’ represents the *Rectified Linear Unit* activation function defined as *f(x) = max(0, x)*, ‘tanh’ represents the hyperbolic tangent activation function defined as *f(x) = tanh(x)*.

**Table 4:** Hyperparameter values associated with the best configurations per model. Model acronyms are listed in the Abbreviations section.

Model Tested	Hyperparameter Values of the Best Configuration per Model
TCN + Attention	n_layers = 3, n_filters = (64, 64, 128), kernel_size = 3, dilation = [1, 2, 4], dropout = 0.15
kNN	n_neighbors = 5, weights = ‘distance’, metric = ‘manhattan’
RF	n_estimators = 137, max_depth = 27, min_samples_split = 4, min_samples_leaf = 3, max_features = ‘sqrt’
Extra Trees	n_estimators = 225, max_depth = 27, min_samples_split = 6, min_samples_leaf = 2, max_features = ‘sqrt’
AdaBoost	n_estimators = 330, learning_rate = 0.037, algorithm = ‘SAMME’
HistGB	max_iter = 275, learning_rate = 0.40, max_depth = 20, l2_regularization = 0.1
XGBoost	n_estimators = 108, learning_rate = 0.16, max_depth = 32, subsample = 0.8
LightGBM	num_leaves = 128, n_estimators = 108, max_depth = 21, learning_rate = 0.40
LR	C = 0.072, penalty = ‘l2’, solver = ‘saga’
RC	solver = ‘lsqr’, alpha = 0.1
MLP	hidden_layer_sizes = (100,50), activation = ‘relu’, learning_rate_init = 0.001

**Table 5:** Mean ± standard deviation (std) of 5-fold cross-validation performance for the best model configurations. Model acronyms are listed in the Abbreviations section.

Model Tested	F_1_ Score ± std	Precision ± std	Recall ± std	Specificity ± std	AUROC ± std	AUPRC ± std	FA/h ± std
TCN + Attention	0.723 ± 0.007	0.704 ± 0.063	0.743 ± 0.037	0.980 ± 0.003	0.868 ± 0.002	0.759 ± 0.004	4.000 ± 0.531
kNN	0.809 ± 0.014	0.869 ± 0.026	0.757 ± 0.009	0.994 ± 0.002	0.996 ± 0.002	0.795 ± 0.026	4.244 ± 1.121
RF	0.978 ± 0.008	0.991 ± 0.009	0.965 ± 0.009	0.999 ± 0.001	0.989 ± 0.010	0.979 ± 0.022	0.729 ± 0.121
Extra Trees	0.954 ± 0.004	0.977 ± 0.014	0.932 ± 0.009	0.999 ± 0.001	0.975 ± 0.002	0.963 ± 0.004	0.909 ± 0.661
AdaBoost	0.805 ± 0.003	0.915 ± 0.005	0.718 ± 0.002	0.997 ± 0.001	0.906 ± 0.001	0.780 ± 0.003	5.403 ± 1.252
HistGB	0.890 ± 0.019	0.952 ± 0.031	0.835 ± 0.021	0.998 ± 0.001	0.962 ± 0.008	0.848 ± 0.053	2.384 ± 1.201
XGBoost	0.863 ± 0.017	0.872 ± 0.045	0.854 ± 0.017	0.999 ± 0.002	0.999 ± 0.004	0.989 ± 0.005	1.390 ± 0.554
LightGBM	0.879 ± 0.017	0.884 ± 0.014	0.875 ± 0.020	0.994 ± 0.001	0.989 ± 0.021	0.955 ± 0.045	3.934 ± 2.101
LR	0.724 ± 0.001	0.679 ± 0.001	0.776 ± 0.001	0.982 ± 0.010	0.911 ± 0.010	0.718 ± 0.001	2.054 ± 4.015
RC	0.773 ± 0.006	0.831 ± 0.059	0.723 ± 0.035	0.993 ± 0.003	0.887 ± 0.003	0.767 ± 0.005	2.003 ± 3.522
MLP	0.882 ± 0.028	0.950 ± 0.049	0.823 ± 0.021	0.998 ± 0.002	0.971 ± 0.007	0.904 ± 0.024	3.608 ± 1.612

**Table 6:** Performance metrics achieved by the best configuration per model, as identified through hyperparameter tuning, evaluated on a hold-out set equal to 15% of the entire dataset. Model acronyms are listed in the Abbreviations section.

Model Tested	F_1_ Score	Precision	Recall	Specificity	AUROC	AUPRC	FA/h
TCN + Attention	0.713	0.704	0.723	0.989	0.876	0.749	3.987
kNN	0.813	0.839	0.789	0.988	0.957	0.919	4.436
RF	0.961	0.975	0.947	0.999	0.999	0.993	0.732
Extra Trees	0.944	0.965	0.924	0.987	0.996	0.994	0.978
AdaBoost	0.812	0.917	0.728	0.988	0.914	0.790	7.609
HistGB	0.877	0.941	0.820	0.979	0.959	0.852	3.135
XGBoost	0.837	0.861	0.815	0.989	0.989	0.998	1.412
LightGBM	0.880	0.899	0.862	0.995	0.983	0.952	3.674
LR	0.719	0.686	0.756	0.989	0.917	0.720	2.032
RC	0.768	0.829	0.715	0.993	0.871	0.773	1.982
MLP	0.781	0.823	0.743	0.998	0.969	0.917	4.513

**Table 7:** Performance metrics of the Random Forest (RF) classifier for different predictive time windows and sequence lengths, evaluated on the 15% test set. Δ*t* is the number of rows between the current and predicted future condition; Δ*t_s_* represents the corresponding prediction horizon, expressed in seconds. *n_s_* indicates the number of consecutive rows available to the model at the time the prediction is made. Rows correspond to measurements acquired every 0.5 s. Metric acronyms are listed in the Abbreviations section.

Δ*t* [-]	Δ*t_s_* [s]	F_1_ Score				Precision				Recall				Specificity			
*n_s_* [-]		10	20	30	40	10	20	30	40	10	20	30	40	10	20	30	40
20	10	0.955	0.957	0.958	0.960	0.974	0.973	0.969	0.960	0.937	0.942	0.948	0.960	0.999	0.999	0.999	0.999
50	25	0.907	0.912	0.917	0.918	0.918	0.924	0.935	0.935	0.897	0.900	0.899	0.901	0.998	0.997	0.996	0.995
80	40	0.877	0.869	0.877	0.878	0.874	0.880	0.879	0.875	0.881	0.859	0.875	0.880	0.997	0.991	0.989	0.990
120	60	0.852	0.852	0.854	0.855	0.831	0.829	0.832	0.831	0.874	0.875	0.879	0.879	0.991	0.977	0.977	0.980
150	75	0.776	0.767	0.771	0.773	0.753	0.756	0.760	0.760	0.801	0.777	0.782	0.787	0.954	0.953	0.961	0.963
**Δ*****t*** **[-]**	**Δ*****t_s_*** **[s]**	**AUROC**				**AUPRC**				**FA/h**				**Selected configuration**			
***n_s_*** **[-]**		**10**	**20**	**30**	**40**	**10**	**20**	**30**	**40**	**10**	**20**	**30**	**40**	**Parameter**			**Case**
20	10	0.999	0.999	0.999	0.995	0.993	0.994	0.993	0.990	0.711	0.691	0.687	0.621	**Δ*****t*** **[-]**			120
50	25	0.999	0.999	0.998	0.998	0.985	0.983	0.980	0.982	1.323	1.234	1.187	1.128				
80	40	0.999	0.997	0.996	0.995	0.988	0.960	0.949	0.952	1.637	1.578	1.432	1.411	**Δ*****t_s_*** **[s]**			60
120	60	0.996	0.993	0.993	0.991	0.939	0.901	0.897	0.899	1.963	1.926	1.957	1.879				
150	75	0.996	0.995	0.994	0.993	0.939	0.913	0.911	0.921	2.733	2.618	2.464	2.198	***n_s_*** **[-]**			10

## Data Availability

The codes presented in this study are openly available on GitHub at the following link: https://github.com/alessianicosia/Treatment_alarm_prediction.git (accessed on 15 September 2026). The dataset used in this study is private and can be accessed upon request.

## Supplementary Materials

The following supporting information can be downloaded at: https://www.mdpi.com/article/10.3390/diagnostics16193095/s1, Table S1. Distribution and functional classification of machine-generated alarm codes included in the binary alarm endpoint; Table S2. Alarm-type-specific event-level performance of the selected Random Forest (RF) configuration (Δ*t* = 120 rows and *n_s_* = 10 rows). Reference [55] is cited in the Supplementary Materials.

## Abbreviations

AdaBoost	Adaptive Boosting
AI	Artificial Intelligence
AUC	Area Under the Curve
AUPRC	Area Under the Precision-Recall Curve
AUROC	Area Under the Receiver Operating Characteristic Curve
AVF	Arteriovenous Fistula
CNN	Convolutional Neural Network
DL	Deep Learning
Extra Trees	Extremely Randomized Trees
FA/h	False Alarms per treatment hour
FPR	False Positive Rate
HD	Hemodialysis
HDF	Hemodiafiltration
HF	Hemofiltration
HistGB	Histogram-based Gradient Boosting
kNN	k-Nearest Neighbors
LightGBM	Light Gradient Boosting Machine
LR	Logistic Regression
MI	Mutual Information
ML	Machine Learning
MLP	Multi-Layer Perceptron
RC	Ridge Classifier
RF	Random Forest
std	standard deviation
TCN	Temporal Convolutional Network
TPR	True Positive Rate
XGBoost	eXtreme Gradient Boosting
