# Supplementary material for: Proactive Prediction of Alarms in Hemodialysis Machines: A Comparative Evaluation of AI Models for Clinical Prognostic Support
Source: Diagnostics (Basel). 2026 Sep 24;16(19):3095. doi: 10.3390/diagnostics16193095 (PMC13664399; doi:10.3390/diagnostics16193095)
Supplement: Supplementary file 1 [file diagnostics-16-03095-s001.zip › diagnostics-4521147-supplementary.pdf]

Supplementary materials

Table S1 provides the complete distribution of the machine-generated alarm codes included in the binary alarm class, reporting both the number of alarm-labeled samples and the number of distinct alarm episodes. The two measures provide complementary information, as multiple consecutive alarm-labeled samples may correspond to a single alarm episode; consequently, a high number of alarm samples does not necessarily indicate a high frequency of independent alarm events.

Alarm codes are additionally grouped according to their functional domain to distinguish technical and operational conditions from treatment-, monitoring-, and safety-related machine alarms. Given the marked prevalence of the "Flowmeter Test" code in **Table S1**, it is worth noting that the flowmeter subsystem of these dialysis machines has already been investigated in a dedicated study published by our research group, reflecting its specific technical relevance and interest to the industrial partner. Further details can be found in Nicosia *et al.* [55].

Table S1. Distribution and functional classification of machine-generated alarm codes included in the binary alarm endpoint. "Alarm samples (n (%))" indicate the number and percentage of individual time samples labeled as alarm, whereas "Alarm episodes (n (%))" represent distinct continuous occurrences of the same alarm code within a treatment session. The "Alarm domain" describes the functional context of each machine-generated alarm, together with the corresponding "Interpretation". Treatment- and safety-related classifications refer to the operational relevance of the alarm within the dialysis procedure and do not imply the occurrence of clinically adjudicated adverse events.

Alarm code	Alarm samples (n (%))	Alarm episodes (n (%))	Alarm domain	Interpretation
Flowmeter Test	103,581 (86.39%)	710 (55.12%)	Technical/ operational	Flowmeter test failure or abnormal flowmeter operation during fluid-delivery monitoring.
Blood Flow Stopped	1,172 (0.98%)	109 (8.46%)	Treatment/ safety-related	Extracorporeal blood-flow interruption or cessation.
Arterial Line Alarm	1,736 (1.45%)	83 (6.44%)	Treatment/ safety-related	Arterial blood-line operating or monitoring abnormality.
Heparin Infusion Off	1,776 (1.48%)	77 (5.98%)	Treatment-related	Abnormal interruption of the administration or delivery of heparin.
End of Ultrafiltration	2,380 (1.99%)	52 (4.04%)	Treatment/ operational	Abnormal interruption of the ultrafiltration phase.
Sphygmomanometer Error – Invalid Measurement	3,784 (3.16%)	50 (3.88%)	Monitoring/ technical	Invalid or unreliable sphygmomanometric blood-pressure measurement.
Pump Off	961 (0.80%)	45 (3.49%)	Treatment/ operational	Unexpected stoppage or deactivation of the monitored pump.

Alarm code	Alarm samples (n (%))	Alarm episodes (n (%))	Alarm domain	Interpretation
Pre-Arterial Pressure Out of Range	80 (0.07%)	28 (2.17%)	Treatment/ safety-related	Pre-arterial blood-line pressure outside the predefined operating range.
Pre-Filter Pressure Out of Range	170 (0.14%)	20 (1.55%)	Treatment/ safety-related	Pre-filter pressure outside the predefined operating range.
Target Weight Loss Not Set	131 (0.11%)	15 (1.16%)	Treatment/ operational	Absence of a configured fluid- or weight-removal target.
Timer Expiration	518 (0.43%)	13 (1.01%)	Operational	Programmed treatment or machine timer timeout.
Sphygmomanometer Error – Aborted Measurement	626 (0.52%)	12 (0.93%)	Monitoring/ technical	Interruption or early termination of blood-pressure measurement before completion.
Pre-Arterial Pressure Minimum/Maximum Limit	17 (0.01%)	12 (0.93%)	Treatment/ safety-related	Pre-arterial pressure outside the predefined minimum/maximum threshold range.
Venous Pressure Out of Range	82 (0.07%)	11 (0.85%)	Treatment/ safety-related	Venous blood-line pressure outside the predefined operating range.
Minimum Ultrafiltration	2,080 (1.73%)	10 (0.78%)	Treatment/ operational	Ultrafiltration level at or below the predefined minimum threshold.
Venous Pressure Minimum/Maximum Limit	23 (0.02%)	10 (0.78%)	Treatment/ safety-related	Venous pressure outside the predefined minimum/maximum threshold range.
Total Dialysate Conductivity	361 (0.30%)	7 (0.54%)	Technical/ safety-related	Abnormal total conductivity of the prepared dialysate mixture.
Arterial Line Connector	24 (0.02%)	4 (0.31%)	Treatment/ safety-related	Arterial blood-line connector or connection-status abnormality.
Battery Alarm	34 (0.03%)	3 (0.23%)	Technical/ operational	Battery or backup-power status abnormality.
Sensor Cover Alarm	11 (0.01%)	3 (0.23%)	Technical/ safety-related	Abnormal cover or accessibility status of the machine sensor section.
Bicarbonate Conductivity	106 (0.09%)	2 (0.16%)	Technical/ safety-related	Abnormal conductivity of the bicarbonate component or bicarbonate preparation stage of the dialysate.
Action on Venous Clamp	83 (0.07%)	2 (0.16%)	Safety-related	Venous-line clamp activation associated with an abnormal blood-return condition.
Air Detected in the Blood Circuit	19 (0.02%)	2 (0.16%)	Safety-related	Presence of air in the extracorporeal blood circuit.
Insufficient Chamber Level	4 (<0.01%)	2 (0.16%)	Treatment/ safety-related	Insufficient fluid or blood level in a monitored chamber.

Alarm code	Alarm samples (n (%))	Alarm episodes (n (%))	Alarm domain	Interpretation
Transmembrane Pressure Minimum/Maximum Limit	80 (0.07%)	1 (0.08%)	Treatment/ safety-related	Transmembrane pressure outside the predefined minimum/maximum threshold range.
Heparin Infusion Alarm	18 (0.02%)	1 (0.08%)	Treatment-related	Heparin infusion or delivery abnormality.
No Water Supply	17 (0.01%)	1 (0.08%)	Technical/ operational	Insufficient or absent water supply for dialysate preparation.
Incorrect Dialysate Mixing	16 (0.01%)	1 (0.08%)	Technical/ safety-related	Abnormal dialysate mixing or composition-control condition.
Venous Line Connector	4 (<0.01%)	1 (0.08%)	Treatment/ safety-related	Venous blood-line connector or connection-status abnormality.
Blood-Side Cover Alarm	2 (<0.01%)	1 (0.08%)	Technical/ safety-related	Abnormal status of the cover or protection element of the blood-side section.
**Total**	**119,896 (100%)**	**1,288 (100%)**		

Table S2 provides the event-level performance of the selected configuration (Δt = 120 rows and n_s = 10 rows), stratified according to the specific alarm code. For each alarm type, the table reports the number of evaluable episodes, the number and proportion of episodes successfully anticipated, and the corresponding mean prediction lead time.

Overall, 154 out of 170 evaluable alarm episodes (90.60%) were successfully anticipated, with a mean lead time of 58.61 s, confirming that the selected Random Forest configuration was generally able to provide warnings close to the targeted one-minute prediction horizon. High detection rates were observed for the most frequent alarm types, including Flowmeter Test (100%, 103/103), Heparin Infusion Off (100%, 7/7), and Blood Flow Stopped (82.35%, 14/17). Conversely, some alarm types were not successfully anticipated at the event level despite correctly classified alarm samples, highlighting differences between sample-level and event-level performance.

Table S2. Alarm-type-specific event-level performance of the selected Random Forest (RF) configuration (Δt = 120 rows and n_s = 10 rows). Here, "Code" identifies the specific machine-generated alarm type; "Alarm samples in test (n)" reports the total number of alarm-labeled samples for each alarm type, while "Detected alarm samples in test (n)" indicates the number of correctly identified alarm samples. "Alarm sample detection rate (%)" represents the percentage of alarm samples correctly detected. "Alarm episodes in test (n)" reports the number of evaluable true alarm episodes of each type, and "Detected episodes (n)" indicates the number of episodes successfully anticipated before alarm onset. "Mean lead time (s)", "Min lead time (s)", and "Max lead time (s)" describe the distribution of the interval between the earliest successful warning and the onset of the corresponding alarm episode. "NA"

stands for "Not Applicable": lead-time statistics are undefined when no alarm episode is detected. Alarm codes listed in **Table S1** but not shown here (Pre-Arterial Pressure Minimum/Maximum Limit, Insufficient Chamber Level, Venous Line Connector, and Blood-Side Cover Alarm) did not occur in the test set

Code	Alarm samples in test (n)	Detected alarm samples in test (n)	Alarm sample detection rate (%)	Alarm episodes in test (n)	Detected episodes (n)	Mean lead time (s)	Min lead time (s)	Max lead time (s)
Flowmeter Test	15,438	13,597	88.07	103	103	58.71	43.50	60.00
Sphygmomanometer Error – Invalid Measurement	583	501	85.93	5	5	59.50	58.00	60.00
End of Ultrafiltration	345	291	84.35	4	4	57.00	54.50	60.00
Minimum Ultrafiltration	300	250	83.33	1	1	60.00	60.00	60.00
Arterial Line Alarm	243	202	83.13	8	0	NA	NA	NA
Heparin Infusion Off	243	199	81.89	7	7	60.00	60.00	60.00
Blood Flow Stopped	190	161	84.74	17	14	60.00	60.00	60.00
Pump Off	142	119	83.80	5	3	58.50	55.50	60.00
Sphygmomanometer Error – Aborted Measurement	101	89	88.12	2	2	56.00	52.00	60.00
Timer Expiration	72	58	80.56	3	2	58.25	56.50	60.00
Total Dialysate Conductivity	57	44	77.19	1	1	38.50	38.50	38.50
Bicarbonate Conductivity	20	17	85.00	1	1	58.00	58.00	58.00
Pre-Filter Pressure Out of Range	18	14	77.78	3	2	60.00	60.00	60.00
Transmembrane Pressure Minimum/Maximum Limit	18	3	16.67	1	1	41.50	41.50	41.50
Target Weight Loss Not Set	18	17	94.44	2	2	55.50	51.00	60.00

Code	Alarm samples in test (n)	Detected alarm samples in test (n)	Alarm sample detection rate (%)	Alarm episodes in test (n)	Detected episodes (n)	Mean lead time (s)	Min lead time (s)	Max lead time (s)
Action on Venous Clamp	15	11	73.33	0	0	NA	NA	NA
Venous Pressure Out of Range	10	8	80.00	1	1	60.00	60.00	60.00
Battery Alarm	8	7	87.50	2	2	60.00	60.00	60.00
Pre-Arterial Pressure Out of Range	8	6	75.00	1	1	60.00	60.00	60.00
Heparin Infusion Alarm	6	0	0.00	0	0	NA	NA	NA
Venous Pressure Minimum/Maximum Limit	6	5	83.33	2	2	60.00	60.00	60.00
Arterial Line Connector	5	4	80.00	1	0	NA	NA	NA
Air Detected in the Blood Circuit	3	2	66.67	0	0	NA	NA	NA
Incorrect Dialysate Mixing	3	0	0.00	0	0	NA	NA	NA
No Water Supply	3	2	66.67	0	0	NA	NA	NA
Sensor Cover Alarm	1	0	0.00	0	0	NA	NA	NA
**Total**	**17,856**	**15,607**	**87.40**	**170**	**154**	**58.61**	**38.50**	**60.00**
